# A new transpedicular lag screw fixation for treatment of unstable Hangman’s fracture: a minimum 2-year follow-up study

**DOI:** 10.1186/s13018-020-01911-3

**Published:** 2020-09-01

**Authors:** Yijie Liu, Yi Zhu, Xuefeng Li, Jie Chen, Sen Yang, Huilin Yang, Weimin Jiang

**Affiliations:** Department of Orthopaedic Surgery, The First Affiliated Hospital of Soochow University, 899 Pinghai Street, Suzhou, 215006 China

**Keywords:** Hangman’s fracture, Transpedicular lag screw, Cervical spine

## Abstract

**Background:**

A new C2 transpedicular lag screw designed by our team has been used in human cadaver spines for biomechanical testing, and the results showed that the biomechanical properties of the new C2 transpedicular lag screw were better than ordinary screws. The objective of this study is to analyze the clinical efficacy and safety of the new C2 transpedicular lag screw fixation for the treatment of unstable Hangman’s fracture.

**Methods:**

From March 2013 to June 2017, 25 patients who had unstable Hangman’s fractures were operated on with a new C2 transpedicular lag screw fixation. The patients included 18 males and 7 females whose ages ranged from 31 to 62 years (average 45.4 ± 9.3 years). The cause of the injury was a traffic accident in 17 patients and a fall from height in 8 patients. Other associated lesions included rupture of the spleen (1 patient) and rib fractures (2 patients). According to the Levine-Edwards classification, 17 patients were type II and 8 patients were type IIA, and according to the Frankel Neurological Performance scale, 8 cases and 17 cases were graded as spinal cord injury D and E, respectively. Twenty-three cases received bilateral screw fixation, and 2 cases had unilateral screw fixation because another pedicle was chipped. The whole procedure was accomplished with monitoring by “C”-arm fluoroscopy.

**Results:**

The mean follow-up time was 36 ± 12 months and ranged from 24 to 60 months. No obvious symptomatic or radiologic postoperative complications were found during the follow-up period. Six cases were restored from D to E while 2 cases remained D according to the American Spinal Injury Association (ASIA) grade. Pre- and postoperative visual analogue scale (VAS) and Neck Disability Index (NDI) were statistically different (*P* < 0.001). Osseous union was achieved in all cases, and the range of cervical motion recovered to the normal level up to the last follow-up.

**Conclusions:**

The primary clinical and radiographic efficacies of a new C2 transpedicular lag screw fixation for the treatment of unstable Hangman’s fracture were satisfactory. This approach could be considered a simple, effective, reliable, and economic surgical method for managing unstable Hangman’s fractures.

## Background

Hangman’s fracture, which is also known as traumatic spondylolisthesis of C2, is defined as a fracture involving the bilateral fracture of the C2 pars interarticularis with a variable displacement of C2 on C3. It is the second most common C2 fracture vertebra and accounting for 4–7% of all cervical spine fractures [1, 2]. The injury was initially introduced by Schneider et al. [3] and is now most commonly caused by motor vehicle accidents and falls. Despite most Hangman’s fractures were treated conservatively, surgery is theoretically preferable in cases of unstable Hangman’s fracture.

In 1964, Leconte [4] described a C2 direct transpedicular fixation for Hangman’s fracture, and it was demonstrated effective by Judet et al. [5]. This fixation of C2, which is considered as a “physiologic operation,” could preserve the motion of normal segments. However, the side effects of a regular C2 transpedicular screw such as incomplete reduction, screw dislodgement, and excessive compression are still unavoidable when direct pars repair is performed. To reduce the potential complications, a new C2 transpedicular lag screw, which was designed by our team, has been gradually applied clinically for Hangman’s fracture. The new C2 transpedicular lag screw has been used in human cadaver spines for biomechanical testing, and the results showed that the biomechanical properties of the new C2 transpedicular lag screw were better than ordinary screws [6]. From March 2013 to June 2015, 25 patients who suffered from unstable Hangman’s fractures were operated on with the new C2 transpedicular lag screw fixation, and all cases achieved satisfactory results. The aim of this study was to report the radiological and clinical outcomes of the new transpedicular lag screw for treating unstable Hangman’s fracture.

## Materials and methods

### Study design and demographic data

This study was approved by the local Medical Ethics Committee (ethical code no. 2020144), and informed consent was obtained from all individual participants included in the study. The inclusion criteria included the following: (1) according to the Levine-Edwards classification, all Hangman’s fractures were type II or type IIA; (2) preoperative cervical MRI indicating no spinal cord compression at the C2–3 level; and (3) no severe osteoporosis. The exclusion criteria included the following: (1) pedicle of C2 developmental malformation, (2) bilateral pedicle comminuted fracture, (3) infection in the operative field, and (4) other severe medical comorbidities that cannot tolerate open surgery (e.g., cardiovascular system diseases, hematological system diseases, severe hepatic and renal dysfunction).

From March 2013 to June 2017, 25 patients with Hangman’s fractures were operated on with a new transpedicular lag screw fixation. There were 18 males and 7 females included in the study with a mean age of 45.4 ± 9.3 years (range 31–62 years). The causes of their injuries were traffic accidents for 17 patients and fallings from height for 8 patients. Other associated lesions included rupture of the spleen (1 patient) and rib fractures (2 patients). According to the Levine-Edwards classification, 17 patients were type II and 8 patients were type IIA. According to the American Spinal Injury Association (ASIA) grade, 8 cases and 17 cases were graded as spinal cord injury D and E, respectively. All patients complained of neck pain and restricted motion of their cervical spine. Overall, 19 of the patients had no neurological deficits, and 6 had numbness in their upper extremities. The patients’ preoperative and postoperative data are shown in Table 1.

**Table 1:** Pre- and postoperative data of 25 patients

Case	Age/sex	Injury	Classification (Levine and Edwards)	Preoperative ASIA grade	Operation time (min)	Estimated blood loss (mm)	Postoperative ASIA grade	Length of stay (days)	Follow-up duration (months)
1	31 years/M	Fall	Type II	E	95	250	E	5	60
2	42 years/F	MVA	Type II	D	100	200	E	5	60
3	32 years/M	MVA	Type IIA	E	88	280	E	5	60
4	43 years/F	Fall	Type IIA	D	130	350	D	6	48
5	47 years/M	MVA	Type II	E	95	250	E	5	48
6	36 years/M	MVA	Type II	E	112	300	E	5	48
7	60 years/F	MVA	Type II	E	115	300	E	5	48
8	40 years/M	MVA	Type IIA	E	123	300	E	5	48
9	37 years/M	Fall	Type IIA	D	120	320	E	7	36
10	52 years/M	MVA	Type II	E	92	350	E	6	36
11	62 years/M	MVA	Type II	E	105	280	E	5	36
12	35 years/F	MVA	Type II	E	80	270	E	5	36
13	44 years/M	Fall	Type IIA	E	118	310	E	5	36
14	52 years/M	MVA	Type II	E	103	290	E	5	36
15	36 years/M	Fall	Type II	E	122	320	E	6	24
16	38 years/F	MVA	Type II	E	96	280	E	5	24
17	43 years/M	MVA	Type IIA	E	125	330	E	5	24
18	46 years/M	MVA	Type II	D	108	290	E	6	24
19	50 years/M	MVA	Type II	E	95	250	E	5	24
20	48 years/M	Fall	Type II	D	110	200	E	5	24
21	62 years/F	MVA	Type IIA	D	83	370	D	6	24
22	57 years/F	Fall	Type IIA	D	92	300	E	7	24
23	43 years/M	Fall	Type II	E	104	250	E	5	24
24	40 years/M	MVA	Type II	D	80	270	E	6	24
25	58 years/M	MVA	Type II	E	95	290	E	5	24

*ASIA* American Spinal Injury Association, *MVA* motor vehicle accident, *M* male, *F* female

### Rationale of the new transpedicular lag screw

**Fig. 1:**
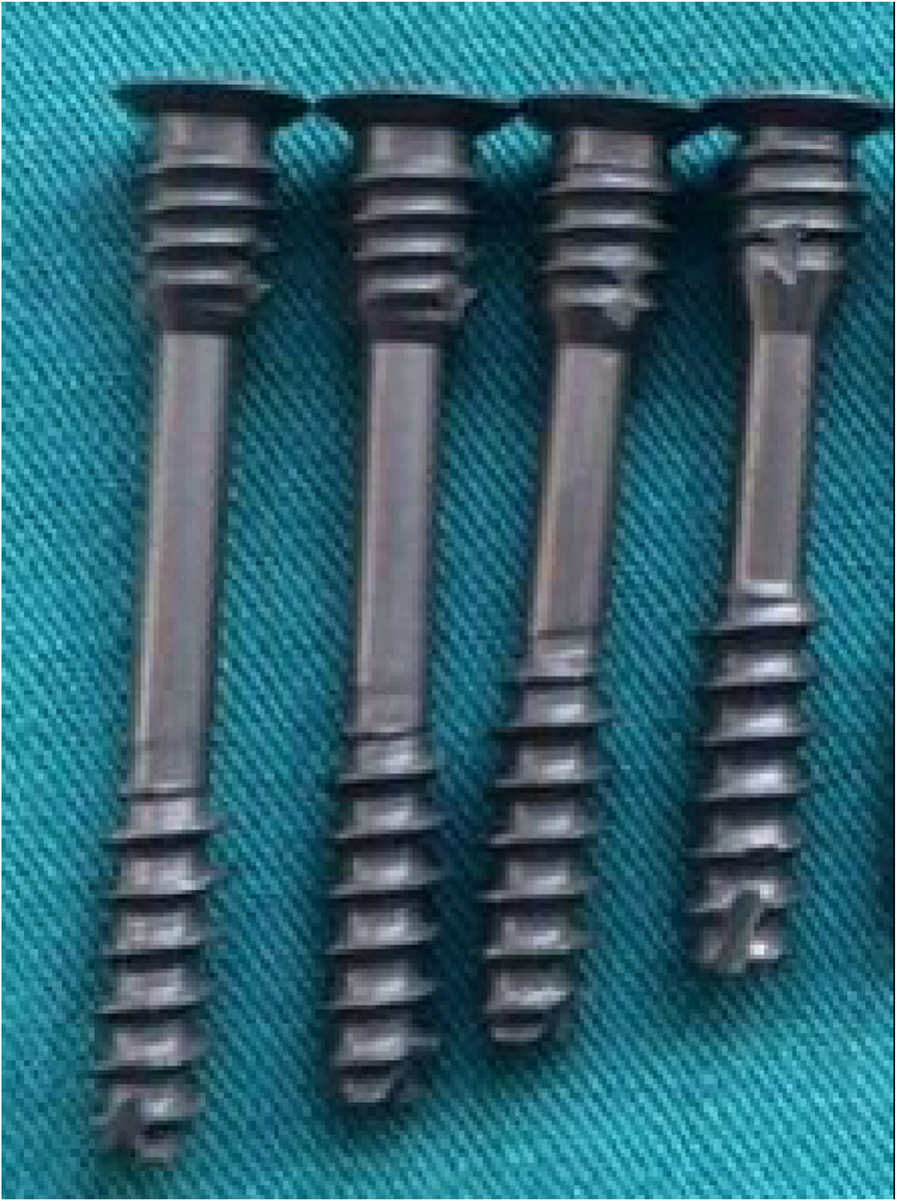
New transpedicular lag screw

The length of the new transpedicular lag screw is 22–32 mm, and the diameter of the head thread is 4 mm. The tail thread is 5 mm, and the screw fastener is 6.4 mm. The pitch of the head thread and tail thread is 1.75 mm and 1.25 mm, respectively. The screw is a double-thread screw and can compress the fracture twice. The head thread passes through the fracture line and links the C2 vertebral body. During the same period, the tail thread of the screw tightened the fractured bone, and the surgeon makes sure that the compression on the posterior part of the pedicle is adequate. This unique structure offers a double-fixation mechanism that is superior to common screws with a single fixation. Furthermore, the tail thread can help avoid excessive compression, which may cause further dislocation. The image of this new transpedicular lag screw is shown in Fig. 1. Other schematic images and details can be seen in our previous article [6].

### Operative procedures and perioperative managements

**Fig. 2:**
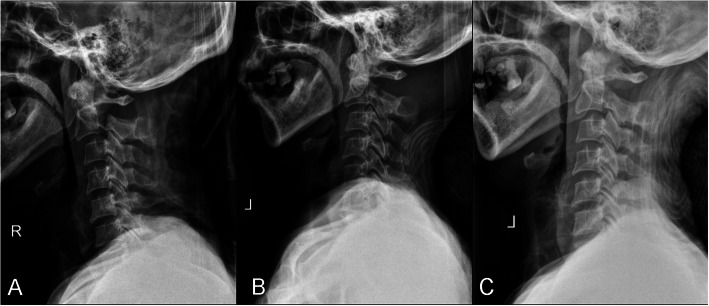
**a** Lateral radiograph, indicating type II Hangman’s fracture. **b** The patient was hospitalized with skull traction, and the lateral radiograph showed the partial reduction after skull traction for 3 days. **c** Lateral radiograph after 5-day skull traction, manifesting complete reduction which represented the fracture end separation was ≤ 2 mm with no obvious angle

All the patients were hospitalized with skull traction. Lateral radiographs were regularly checked to adjust the traction weight and angle according to the traction effect and reset condition. The traction managed to reduce dislocation completely in 16 patients whose fracture end separations were ≤ 2 mm with no obvious angle (Fig. 2), while partially in the other 9 patients. The radiographic assessment included preoperative standard anteroposterior, lateral, and open-mouth views of the cervical spine, and computed tomography (CT) reconstructions were obtained in each patient. Magnetic resonance imaging (MRI) was performed to exclude spinal cord compression and evaluate the integrity of the C2–3 intervertebral disc.

**Fig. 3:**
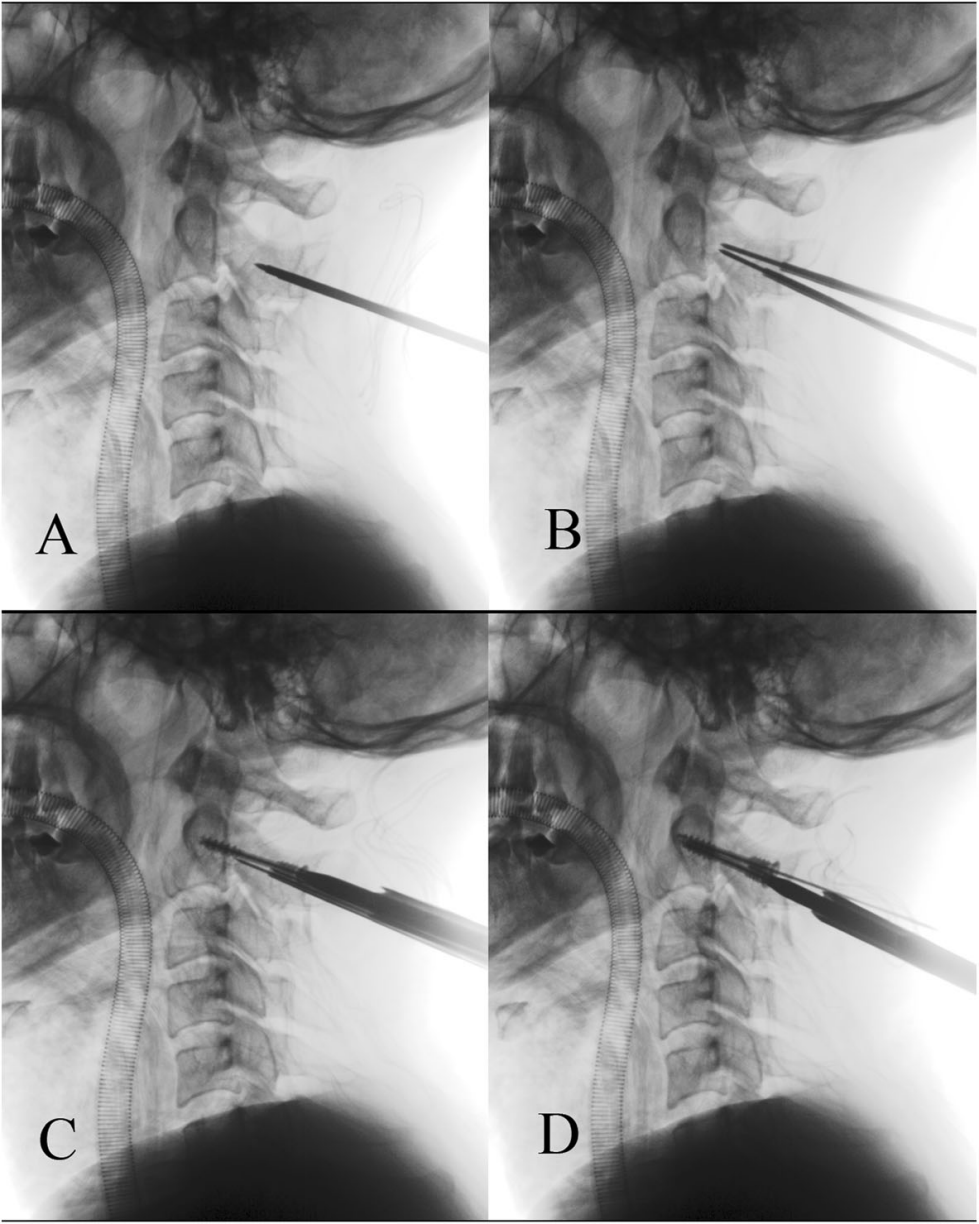
Intraoperative images of C-arm radiographs. **a** Confirm the entry point and the direction of the screw. **b** Insert the guide pin through the fracture line and then drill a hole by the drill bit along the direction of the guide pin. **c**, **d** Screw appropriate-sized new transpedicular lag screws on either side by passing through the drill hole and achieve firm compression and fixation

All surgeries were performed by the same experienced surgeon. Patients received general anesthesia and were placed in the prone position. Under image control, the initial reduction was achieved by placing the head in a slightly flexed position and keeping the skull in traction. A standard midline incision was made above the C1–C3. At the level of C2, the lateral margins of the inferior articular processes were exposed bilaterally to precisely locate the point of entry of the screw at the entrance of the posterior aspect of the lateral mass. The drill bit was parallel to both the medial and superior border of C2 pars interarticularis (usually 15–20° cephalad to the transverse plane and 25–30° medial to the sagittal plane). The drill hole passed through the posterior part of the pedicle, fracture site, and the true pedicle then stopped anterior within the C2 vertebral body. An appropriate-sized new transpedicular lag screw was placed on either side by passing through the drill hole. The final reduction and positions of the screws were confirmed by an image intensifier in the lateral and AP views. Overall, 24 cases received bilateral screw fixation, and one case received unilateral screw fixation because another pedicle was chipped. Intraoperative images are shown in Fig. 3.

Routine closure was performed, and drains were left in place as needed for 24–48 h. All patients had prophylactic antibiotic coverage as well as dexamethasone and mannitol for 3 days. Patients were asked to sit up 3 days after and walk with a neck collar within 2 weeks. If neck pain still exists and the visual analogue scale (VAS) score is over 4, neck collar is required for another 2 weeks. VAS scores and Neck Disability Index (NDI) were collected pre- and postoperatively. Postoperative radiograph and CT were obtained before the patient was discharged from the hospital, and the patients were routinely examined at 3 months, 6 months, and every year after the operation. Follow-up clinical examinations were obtained by the same physician of our team.

### Statistics

Data entry and statistical analysis were performed using the Statistical Package for the Social Sciences software (version 20.0, SPSS, USA). The measurement data are expressed as mean ± SD. Comparisons of clinical and radiological outcomes pre- and postoperatively were performed using a paired *t* test. Differences were considered statistically significant when *P* < 0.05.

## Results

**Fig. 4:**
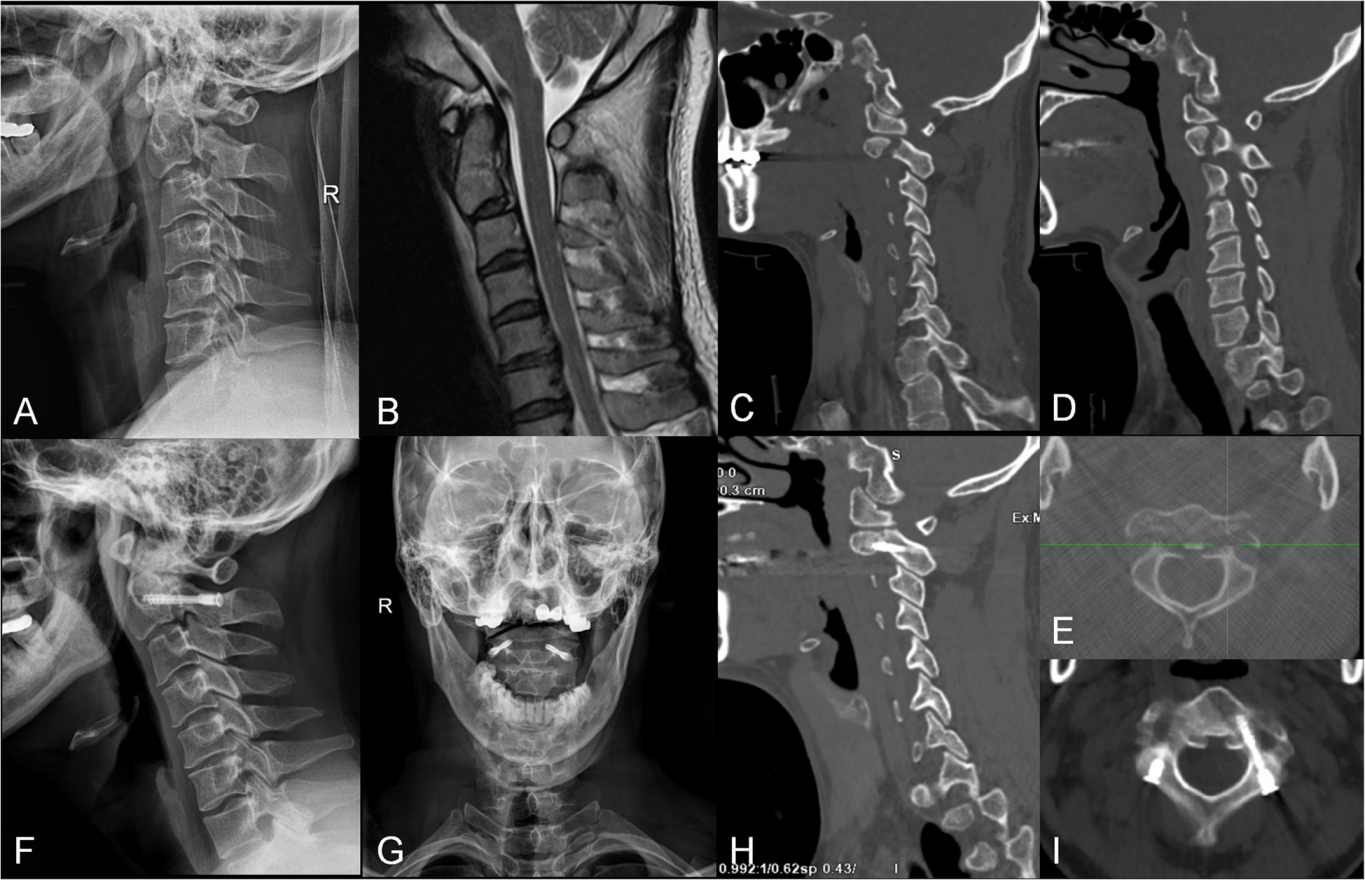
**a** The preoperative lateral radiograph of a 32-year-old male patient with a type IIA Hangman’s fracture. **b** A preoperative sagittal T2-weighted magnetic resonance image (MRI) revealed the absence of spinal cord compression. **c**–**e** The preoperative computed tomography (CT) scan reconstructions showed an obvious separation of fracture on both sides. **f**, **g** The postoperative lateral and open-mouth radiographs showed adequate fracture reduction. **h**, **i** The CT scan reconstructions at the 3-month follow-up showed satisfied osseous union

All the surgeries were successful, and all patients were followed up at least 24 months with a mean follow-up period of 36 ± 12 months (range 24–60 months). The average operation time was 103.4 ± 14.5 min (range 80–130 min), the estimated blood loss was 288.0 ± 41.5 ml (range 200–350 ml), and the average LOS was 5.4 ± 0.6 days (range 5–7 days) (Table 1). Compared with preoperative scores, the postoperative VAS scores and NDI were statistically significantly improved (Table 2). There were no spinal cord or vertebral artery injuries intraoperatively. No screws became loose or broke, and there were no cervical malformations or instability during the follow-up period. The only complication observed was one case of incision infection which recovered after dressing and antibiotics intravenously guttae. According to the ASIA grade, 6 cases were restored from D to E while only 2 cases remained D. The other 17 cases were still all in grade E. Osseous union was achieved for all the cases. Pre- and postoperative images of a typical patient are shown in Fig. 4.

**Table 2:** Summarization of the VAS scores and NDI (mean ± SD)

Parameters	VAS scores	*P* value	NDI	*P* value
Preoperative	8.0 ± 0.9		0.85 ± 0.07	
Postoperative 1 day	3.9 ± 1.0^a^	< 0.001	0.64 ± 0.12	< 0.001
Postoperative 1 month	1.5 ± 0.7^a^	< 0.001	0.48 ± 0.08	< 0.001
Postoperative 3 months	1.4 ± 0.8^a^	< 0.001	0.17 ± 0.04	< 0.001
Final follow-up	1.3 ± 0.7^b^	0.60	0.16 ± 0.04	0.31

*VAS* visual analogue scale, *NDI* Neck Disability Index

^a^*P* compared with preoperative value

^b^*P* compared with postoperative 3 months

There were significant differences in cervical range of motion between 3 months follow-up and 6 months follow-up, considering that some cases (8/25) had not achieved complete osseous union in 3 months follow-up, but this condition remarkably improved during the next 3 months, so that the cervical range of motion was significantly improved in 6 months follow-up (*P* < 0.001, Table 3). At the last follow-up, the cervical range of motion showed no statistical difference from that at 6 months follow-up, indicating that all patients had no loss of cervical range of motion (*P* > 0.05, Table 3).

**Table 3:** The mean cervical activity measured during follow-up (mean ± SD)

Parameters	Flexion	Extension	Left flexion	Right flexion	Left rotation	Right rotation
Postoperative 3 months	24.7 ± 3.2	25.9 ± 2.7	24.4 ± 4.3	24.9 ± 3.8	51.2 ± 6.2	50.7 ± 5.8
Postoperative 6 months	37.5 ± 2.0^a^	39.3 ± 1.8^a^	39.2 ± 2.1^a^	40.1 ± 2.0^a^	70.6 ± 2.6^a^	69.8 ± 2.5^a^
Final follow-up	37.9 ± 1.8^b^	40.0 ± 1.5^b^	40.1 ± 1.3^b^	40.9 ± 1.7^b^	70.8 ± 2.1^b^	70.4 ± 1.3^b^
^a^*P*	< 0.001	< 0.001	< 0.001	< 0.001	< 0.001	< 0.001
^b^*P*	0.25	0.23	0.08	0.15	0.58	0.33

^a^*P* < 0.05 compared with postoperative 3 months

^b^*P >* 0.05 compared with postoperative 6 months

## Discussion

Hangman’s fracture was initially described in 1965 and is the most frequent upper cervical fracture after the odontoid fracture. However, optimal treatment for Hangman’s fracture is still unclear. The fractures are classified based on the classification proposed by Effendi et al. [7] and modified by Levine and Edwards [8]. Type I has stable and minimal translation (< 2 mm) without C2–C3 angulation, type II has C2–C3 angulation and translation (> 2 mm), type IIA is unstable due to flexion-distraction injury and has more angulation without translation, and type III is unstable and has severe C2–C3 angulation and translation.

In most cases of type I Hangman’s fractures, the conservative treatment is used the most [9, 10]. However, the halo or traction device immobilizing time and the possibility of pseudarthrosis, anterior dislocation, and kyphosis suggest that surgical treatment might be a good option. Surgical stabilization is recommended for Levine-Edwards type II, type IIA, and type III fractures with obvious dislocation [11–13]. The treatment goals in Hangman’s fracture are to achieve anatomical reduction, maintain alignment, and maintain the patients’ ability to have an active life. Different surgical approaches, both anterior and posterior, have been described for treating Hangman’s fracture [14, 15]. An anterior approach has the advantage of a technically simple and relatively short fusion construct involving a C2–C3 discectomy with interbody fusion and plating [16, 17]. The anterior approach, however, cannot address the detached posterior arch of C2 and may have approach-related problems. The high risks of anterior approach were mainly embodied in injuries to vital structures, especially in the facial and hypoglossal nerves, branches of the external carotid artery, contents of the carotid sheath, and the superior laryngeal nerve [18, 19]. The posterior approach was associated with a relatively simple exposure with no major vascular and visceral structures as well as a lower complication rate. However, both the anterior cervical discectomy and fusion (ACDF) and posterior C1–C2 or C2–C3 screw fixation will lose mobility of the fused segment. Direct repair of the pars fracture with a transpedicular screw across the fracture line has the advantage of preserving motion of the axis [16, 20, 21] and is recognized as a “physiologic operation.” Borne et al. [21] reported a direct transpedicular screw fixation in 13 cases of Hangman’s fractures, and all the patients had excellent results. ElMiligui et al. [20] also performed the operation in 15 patients and found it to be a simple and safe method. However, traditional transpedicular screw fixation for Hangman’s fracture has several disadvantages. First, the reduction cannot be easily achieved with a traditional transpedicular screw because the direction of the screw hole is not usually perpendicular to the fracture line, which may cause loss of reduction during the compression. Second, it could not offer sufficient stability because the traditional transpedicular screw has only head thread. Third, this approach easily causes excessive compression, and the amplitude of compression relies on the surgeon’s experience.

This new transpedicular lag screw is a double-thread screw based on a Herbert screw and can compress the fracture twice. The diameter of the head thread is less than the tail thread, and the corresponding pitch of the head thread is longer than the pitch of the tail thread. This design offers finite compression that could avoid the loss of reduction in fractures. Compared to the traditional transpedicular screw, this lag screw is associated with significant benefits: (1) it is a biomechanically strong repair method with an unequal pitch double-thread and can help to decrease stress shielding and increase osseous union; (2) it is a safe operation because it can avoid excessive compression to some degree through the surgeon’s feeling when screwing; and (3) it can shorten the hospital stay and allow for early rehabilitation with a better quality of life since patients would receive early mobilization with a neck collar 3 days postoperatively and without neck collar 2 weeks postoperatively due to the firm fixation. In our study, the results showed the new transpedicular lag screw fixation was effective for treating Hangman’s fracture. There were no infections or hemorrhages, and the postoperative CT showed there were no instances of loose screws or ruptures. All patients obtained excellent osseous union and good stability by the end of their follow-up period.

Although the direct transpedicular lag screw fixation for Hangman’s fracture is considered to be physiologic reconstruction and has been advocated for, it is not appropriate for all types of Hangman’s fractures. According to the Levine-Edwards classification, type I, type II, and type IIA after reduction with skull traction can be performed with transpedicular lag screw fixation. It is not advised to manage the fractures with direct C2 transpedicular lag screw fixation if there was excessive disc and ligament damage. As such, MRI was performed to exclude spinal cord compression and evaluation of the integrity of the C2–C3 intervertebral disc preoperatively. In addition, a type III Hangman’s fracture combined with bilateral facet dislocation is not indicated for the method. For this instance, we advocate a posterior C2–C3 screw technique.

Direct transpedicular lag screw fixation is technically difficult because of the large individual variation in the pedicle dimensions and the course of the vertebral artery. Therefore, the successful placement of cervical pedicle screws requires a 3-dimensional knowledge of the pedicle morphology to identify an ideal screw axis accurately and to avoid neurovascular injury [17, 22]. Accordingly, the rate of injury to vital structures varied between 11 and 66% [23, 24], which motivated adequate preoperative examination. CT scanning with 3-dimensional reconstruction or a MRI evaluation of the spine is essential for detecting individual variations in the dimensions of the pedicle before surgery. Furthermore, all the surgeries were carried out under fluoroscopy, which allowed for accurate intraoperative control of instruments and implant placement, determination of appropriate screw length, anatomical fracture reduction, and anchoring of the screw tip in the opposite cortex. Finally, this technique requires thorough knowledge of spinal anatomy and a great deal of experience in subaxial cervical surgery. Our clinical results suggest that the trajectory guide towards the C2 vertebral body should maintain more inclination inwards and upwards in the axial and sagittal planes. In our study, all the patients regained satisfactory functional outcomes with no limitation of motion and obtained excellent osseous union as well as good stability by their last follow-up.

Our study has several limitations. First of all, the number of cases in this study is small. Experience with a greater number of patients and long-term follow-up is still necessary to further evaluate this technique. Secondly, this study is an uncontrolled case series which only manifested the feasibility, safety, and effectiveness of a new C2 transpedicular lag screw fixation for the treatment of unstable Hangman’s fracture. Further controlled prospective studies comparing our lag screw with ordinary screws and conservative treatment are needed.

## Conclusions

The primary clinical and radiographic efficacies of a new C2 transpedicular lag screw fixation for treating Hangman’s fracture were satisfactory. This approach involves a simple operation, small invasive, lower expenses, and a rigid fixation. As such, this approach could be considered a simple, effective, reliable, and economic surgical method for treating unstable Hangman’s fractures.

## Data Availability

The datasets used and/or analyzed during the current study are available from the corresponding author on reasonable request.

## Abbreviations

ASIA: American Spinal Injury Association
CT: Computed tomography
MRI: Magnetic resonance imaging
ACDF: Anterior cervical discectomy and fusion
